# The effect of refurbishing a UK steel plant on PM_10 _metal composition and ability to induce inflammation

**DOI:** 10.1186/1465-9921-6-43

**Published:** 2005-05-18

**Authors:** Gary R Hutchison, David M Brown, Leon R Hibbs, Mathew R Heal, Ken Donaldson, Robert L Maynard, Michelle Monaghan, Andy Nicholl, Vicki Stone

**Affiliations:** 1 Biomedicine Research Group, Napier University, Edinburgh EH10 5DT, UK; 2 School of Chemistry, University of Edinburgh, West Mains Road, Edinburgh, UK; 3 ELEGI & COLT Research Laboratory, Medical School, University of Edinburgh, UK; 4 Department of Health UK, Skipton House, 80 London Road, London SE1 6LH, UK; 5 Institute of Occupational Medicine, Research Park North, Riccarton, Edinburgh, EH14 4AP, Scotland, UK

## Abstract

**Background:**

In the year 2000 Corus closed its steel plant operations in Redcar, NE of England temporarily for refurbishment of its blast furnace. This study investigates the impact of the closure on the chemical composition and biological activity of PM_10 _collected in the vicinity of the steel plant.

**Methods:**

The metal content of PM_10 _samples collected before during and after the closure was measured by ICP-MS in order to ascertain whether there was any significant alteration in PM_10 _composition during the steel plant closure. Biological activity was assessed by instillation of 24 hr PM_10 _samples into male Wistar rats for 18 hr (n = 6). Inflammation was identified by the cellular and biochemical profile of the bronchoalveolar lavage fluid. Metal chelation of PM_10 _samples was conducted using Chelex beads prior to treatment of macrophage cell line, J774, *in vitro *and assessment of pro-inflammatory cytokine expression.

**Results:**

The total metal content of PM_10 _collected before and during the closure period were similar, but on reopening of the steel plant there was a significant 3-fold increase (p < 0.05) compared with the closure and pre-closure samples. Wind direction prior to the closure was predominantly from the north, compared to south westerly during the closure and re-opened periods. Of metals analysed, iron was most abundant in the total and acid extract, while zinc was the most prevalent metal in the water-soluble fraction. Elevated markers of inflammation included a significant increase (p < 0.01) in neutrophil cell numbers in the bronchoalveolar lavage of rats instilled with PM_10 _collected during the reopened period, as well as significant increases in albumin (p < 0.05). Extracts of PM_10 _from the pre-closure and closure periods did not induce any significant alterations in inflammation or lung damage. The soluble and insoluble extractable PM_10 _components washed from the reopened period both induced a significant increase in neutrophil cell number (p < 0.05) when compared to the control, and these increases when added together approximately equalled the inflammation induced by the whole sample. PM_10 _from the re-opened period stimulated J774 macrophages to generate TNF-α protein and this was significantly prevented by chelating the metal content of the PM_10 _prior to addition to the cells.

**Conclusion:**

PM_10_-induced inflammation in the rat lung was related to the concentration of metals in the PM_10 _samples tested, and activity was found in both the soluble and insoluble fractions of the particulate pollutant.

## Introduction

Elevated levels of ambient respirable particulate matter (PM_10_) are associated with increased morbidity and mortality, especially in susceptible individuals [1]. The composition of PM_10 _is variable and complex, which makes identification of the toxic material all the harder, although a variety of components have been proposed to induce inflammation leading to adverse health effects [2].

**Figure 1:**
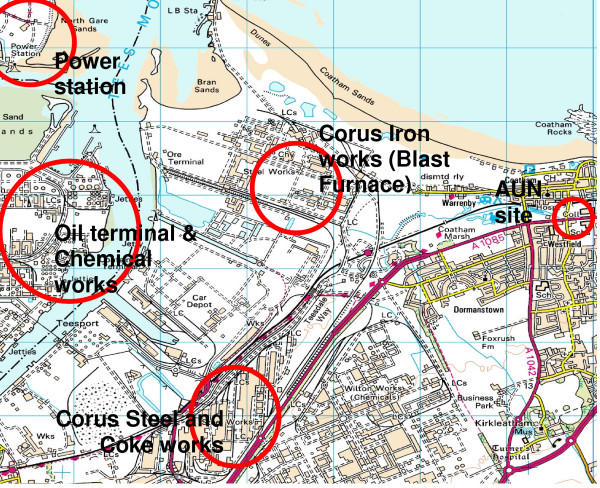
Map of Redcar and surrounding industrial sites. AUN TEOM collection site in proximity to the blast furnace.

In 2000 the steel plant located at the Teesside works in Redcar, UK closed temporarily for a major repair programme to its blast furnace. During this period all steel making and casting operations at Lackenby and ore sintering at Redcar ceased (figure 1). The Department for Environment, Food and Rural Affairs (Defra) and the Devolved Administrations took advantage of this refurbishment to investigate the effect that closing the plant would have on locally produced PM_10. _

This is the first study of its kind in the UK, but is similar in concept to that of the Utah study by Pope [1] Pope *et al*.,[3] reported that, during the closure of a steel mill in the Utah valley, a reduction in PM_10 _mass, and changes in its composition were associated with decreases in morbidity and mortality of the local population. The Utah scenario was a landmark study as it is unusual for an environmental intervention study to take place where the major source of the pollution is closed off and switched on again, allowing researchers to examine clearly the effects of air pollution. The temporary closure of the Utah valley steel mill provided researchers with the unique opportunity to demonstrate a correlation between changes in PM_10 _composition and observed health outcomes. Alterations were observed in PM_10 _composition and mass during the closure period [4]. Changes in total mass did not account for all of the variation in the biological effects of PM_10 _in the Utah valley between the closure of the steel mill, during its shutdown and following its reopening [4]. The hypothesis put forward suggested that the metal component of the PM_10 _was the predominant factor in driving inflammation. Workers at the US Environmental Protection Agency (EPA) showed the importance of the metal content of the Utah valley PM_10 _in relation to its toxicity and pro-inflammatory potential, by carrying out a range of human [4], animal [5] and *in vitro *studies [6,7]. Further analysis of Utah PM_10 _metal content showed iron (Fe), copper (Cu) and zinc (Zn) to be abundant during the active periods of the steel mill, but to be substantially reduced during closure. Such transition metals can act as initiators of inflammation and cytotoxicity via oxidative mechanisms, such as redox cycling. It has also been hypothesised that the allergen or endotoxin content of the PM_10_may have a role with respect to effects on health. None of these hypotheses have yet been proven, but the case for the role of transition metals has been emphasised through research into the Utah episode.

The current study aimed to investigate whether closure of a UK steel plant blast furnace would also impact upon the metal content of PM_10 _and whether this change in composition would alter the biological potency of this pollutant.

## Methods

All materials were obtained from Sigma (Poole, U.K.) unless otherwise stated.

### PM_10 _sample collection

PM_10 _samples were collected by Redcar and Cleveland Council, in collaboration with Casella Stanger using a Tapered Element Oscillating Microbalance (TEOM) with Automated Cartridge Collection Unit (ACCU). The flow rate was 16.7 l/min equivalent to the human lung ventilation rate. This means that over 24 hours 24048 l of air were sampled, and each filter was used to collect PM_10 _for 6–8 days. The sampling location was the Redcar Automated Urban Network (AUN) site, to the east of the steel plant blast furnace in a highly populated area (figure 1). Samples were collected from 21/06/00 until 15/12/00, during which time the steel plant closed operations on the week commencing 26/07/00 and reopened 28/09/00. At this location, the Corus Teesside works is the major industrial source of PM_10 _(table 1). To conduct compositional and toxicological analysis, PM_10 _filters were randomly selected from each of the 3 periods.

**Table 1:** Environment agency PM_10 _emissions data collected from year 2000 within the Redcar area (* Corus operations effected by blast furnace relining shown in map figure 1) [8].

**REDCAR PM_10 _RELEASE FROM LOCAL INDUSTRY**		
**OPERATOR NAME**	**SITE ADDRESS**	**TOTAL RELEASED (tonnes)**

CORUS UK LTD	TEESSIDE IRON WORKS (Blast furnace)*	1496
CORUS UK LTD	STEEL HOUSE*	55
CORUS UK LTD	TEESSIDE COKE WORKS*	33
CORUS UK LTD	TEESSIDE TECHNOLOGY CENTR	<1
	WILTON POWER STATION	<1
HECKETT MULTISERV	BRITISH STEEL PLC (SR) LTD TEESSIDE WORKS	<1
HECKETT MULTISERV (SR) LTD	TEESSIDE WORKS	<1
HECKETT MULTISERV (UK) LTD	SURFACE DRESSING	<1
HUNTSMAN POLYURETHANES (UK) LTD	ANILINE PLANT	<1

### Wind rose construction

The wind speed and direction data obtained from Redcar and Cleveland Council and the Meteorological Office allowed the construction of wind roses for the town of Redcar centred at the AUN site. Four roses were constructed to examine the effect, if any, of wind speed and direction on particulate matter: (a) before the closure, (b) during the closure (c) on reopening of the plant and (d) the entire sampling period.

### Chemical compositional analysis

**Figure 2:**
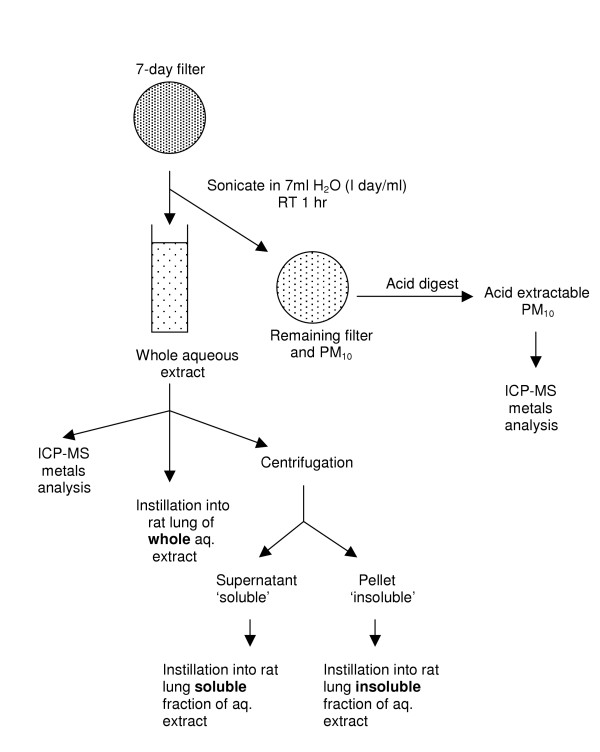
Diagram detailing the methods used to prepare samples to examine composition and toxicity of Redcar PM_10_.

A schematic of the extraction methodology is shown in figure 2 and followed that reported in detail by Heal [9]. The water extractable component of the PM_10 _samples was obtained by sonicating one filter that had been used to sample PM_10 _for 6–8 days in 6–8 ml of 18 Mohm water (i.e. 1 ml/24 hrs of PM_10_) at room temperature for 1 hr to generate suspension of dissolved and insoluble substances. Blank filters were also extracted using the same procedure for comparison. The PM_10 _components remaining on the filter were extracted by subsequent acid digestion using 2.8:1 HCl: HNO_3 _and sequentially heated and evaporated to dryness over 24 hrs. Both the aqueous extract and the acid extract samples were re-suspended in 2% HNO_3 _for analysis. The metal composition of the aqueous and acid extract PM_10 _samples was determined by inductively coupled plasma mass spectrometry (ICP-MS) to quantify the trace metal content of PM_10_. The elements measured were iron, zinc, copper, manganese, cobalt, nickel, chromium, vanadium, titanium, lead, arsenic and cadmium. Total metals as reported here refer to the arithmetical sum of the concentrations of these measured metals. The samples analysed for metal content are described in Table 2, these samples were also used for instillation into rats.

**Table 2:** PM_10 _samples analysed for metal content and then subsequently instilled into rats. PM_10 _was collected using a TEOM ACCU with a flow rate of 16.7 l/min.

**Filter Dates**	**Period of collection (days)**	**Mass of PM_10 _collected onto filter (μg)**	**PM_10 _collected per 24 hours (μg)**	**Maximum PM_10 _dose instilled (μg)**
21/6–29/6	8	2893	360	180
29/6–6/7	7	2186	312	156

26/7–3/8	8	3741	466	233
1/9–7/9	6	3071	510	255

5/10–12/10	7	2011	286	143
26/10–2/11	7	1569	224	112

### Intratracheal instillation of aqueous extracts of Redcar PM_10_

Male Wistar rats (Charles River UK LtD Manson Road Kent) were housed under standard conditions (Rats were kept between 20–22°C 4 per cage in a 12 hour light 12 hour dark cycle cages, bottles and food were changed and washed weekly). Rats weighed between 250 and 300 g at time of use (approximately 3 months old). Three rats were used for each treatment group and there were four treatment groups in total. Ethical approval for this project was obtained via the University Ethics committee.

Group one consisted of animals exposed to saline only (control), group 2 were treated with pre-closure PM_10 _extracts, group 3 with the closure extracts and group 4 with extracts collected on reopening of the steel plant. Each rat received the same aqueous extract of PM_10 _used in the metals analysis described in table 2. Saline was added to extracts prior to instillation to ensure the treatment was at physiological salt concentration. The PM_10 _dose given was not equalised for mass, but was the equivalent of a 24-hour PM_10 _exposure (table 2). It should be stressed that the values provided in table 2 are estimates based upon the flow rate of the sampler, and the ambient PM_10 _concentrations reported at the AUN site during the periods of collection for each filter. On this basis, the maximum PM_10 _dose instilled assumes a 100% efficiency for the recovery of PM_10 _from the filter. However, this is not the case. It was not possible to determine the efficiency of recovery by spectrophotometry due to the low turbidity of the samples recovered. Furthermore, it was not possible to reweigh the filters after extraction since the filters were digested by acid to extract the remaining metal on the filter.

The experiment was subsequently repeated after dividing the aqueous PM_10 _extract into soluble and insoluble extractable PM_10 _components. These samples were prepared from the aqueous PM_10 _washed from filters using water as described previously (1 ml water per 24 hours of PM_10 _collection), this extract was then separated into the soluble and insoluble fractions by centrifugation (12000 g). The insoluble pellet was resuspended in water (again 1 ml per 24 hours of PM_10 _collection). Both samples were treated with saline to generate a physiological salt concentration before subsequently instilling 0.5 ml into each male Wistar rat.

Rats were anaesthetised with halothane and then instilled intratracheally with 500 μl of treatments. As previously described 24 hr PM_10 _was extracted into 1 ml of water, however the exact concentrations of PM_10 _dose are unknown, as turbidomitry could not be carried out due to the low particle concentration and clarity of samples. The figures in table 2 represent the quantity of PM_10 _collected on each filter per 24 hr, but since recovery from the filter is less than 100% and each animal receives 500 μl, these figures are far greater than the dose administered. At 18 hrs following instillation the rats were euthanised by intraperitoneal injection of Euthatal and the lungs surgically removed. Eight ml of saline was injected into the lungs through a cannula and the lobes were massaged for 2 minutes to remove migratory cells and lung lining fluid. This primary bronchoalveolar lavage (BAL) fluid, removed from the lungs was kept separated from three further lavages, 8 ml each, which were pooled to form a secondary lavage. The primary lavage was kept separate from the secondary lavage in order to minimise dilution of constituents. After centrifugation (900 g for 2 minutes) the cells were re-suspended in 1 ml sterile saline and the cells from the primary and secondary lavage samples were pooled. A total cell count was determined, followed by cytospot preparations. These were stained with Diff Quick (Lamb) before determination of differential cell counts.

### BAL biochemical analysis

The primary BAL from each rat was analysed for markers of cellular and tissue damage including lactate dehydrogenase (LDH) activity, [10,11] total protein [12] and albumin protein (bromocresyl green) levels. The pro-inflammatory cytokine proteins, tumour necrosis factor α (TNFα) and macrophage inflammatory protein 2 (MIP2) was also measured by enzyme-linked immunosorbent assay (ELISA) according to the manufacturer's guidelines, Biosource UK Cytosets™.

### Assessment of pro-inflammatory cytokine mRNA expression in BAL cells using Multiprimer PCR

The BAL cells recovered from the control and treated animals were centrifuged (900 g, 2 min) and the pellet washed with phosphate buffered saline (PBS) before addition of 200 μl of Tri-reagent to the cells. The mixture was incubated for 10 minutes at 4°C, and stored at -80°C until required.

The mRNA purification and synthesis of cDNA was carried out following protocols provided with the Biosource Cytoxpress kit™. The human inflammatory cytokine Multiprimer PCR kit from Biosource was used to assess the mRNA expression of 6 cytokines (TNFα, transforming growth factor beta (TGFβ), MIP2, interleukin 6 (IL6), interleukin 1 beta (IL1β), granulocyte macrophage colony stimulating factor (GM-CSF) and 1 housekeeper gene (glyceraldehyde 3-phosphate dehydrogenase, GAPDH) according to the manufacturers guidelines.

The PCR products were detected and quantified by electrophoresis using a 1.5% agarose gel, in a horizontal Biorad GT system. The gels were stained with ethidium bromide and PCR products were detected using a UV transilluminator. Images were taken under UV conditions using a Synygene camera and the intensities of PCR product bands were quantified using Syngene software and expressed as a percentage of the house keeping gene (GAPDH) and then as a percentage of the negative control.

### The effect of removal of Redcar PM_10 _metals via chelation experiments

The murine macrophage cell line, J774.1A was cultured in RPMI 1640 medium containing 10% heat inactivated foetal bovine serum (FBS), 1% L-glutamine, 0.06 U/ml penicillin, 30 mg/ml streptomycin, (all obtained from Life Technologies). The cells were grown and sub-cultured under standard conditions. Cells were removed from flasks using sterile cell scrapers (SLS, UK). J774.1A macrophage cells were treated with samples of Redcar PM_10 _for 4 hrs. Along side these treatments cells were treated with Redcar PM_10 _samples that had under gone chelation to remove metals. This was carried out by suspending particles in RPMI-1640 containing 50 mg/ml chelex beads and mixed on a rotating wheel for 4 hrs at room temperature. After incubation, samples were centrifuged at 12000 g (5 min) to pellet the chelex beads. The resultant suspensions were applied to J774.A1 cells and incubated at 37°C for 4 hrs. Cell culture supernatants were subsequently analysed for TNFα protein via ELISA (Biosource UK Cytosets™).

### Statistical analysis

Experiments were conducted, at minimum, in triplicate and the data shown in each figure represents the mean of three separate experiments ± the standard error of the mean (S.E.M) unless other wise stated. Statistical significance was determined using One Way Analysis of Variance (ANOVA) with Tukey's pair wise comparison (Minitab Version 13). * p < 0.05 is denoted as being significant, with ***p < 0.001 representing high significance.

## Results

### Redcar PM_10_, wind speed and direction before, during and after blast furnace closure

The PM_10 _mass collected per 24 was greater during the closure period than in the preclosure or postclosure periods (Table 2). There is no information available to explain this observation, however coarse particulate emissions may have been increased during refurbishment and repair of the blast furnace lining.

**Figure 3:**
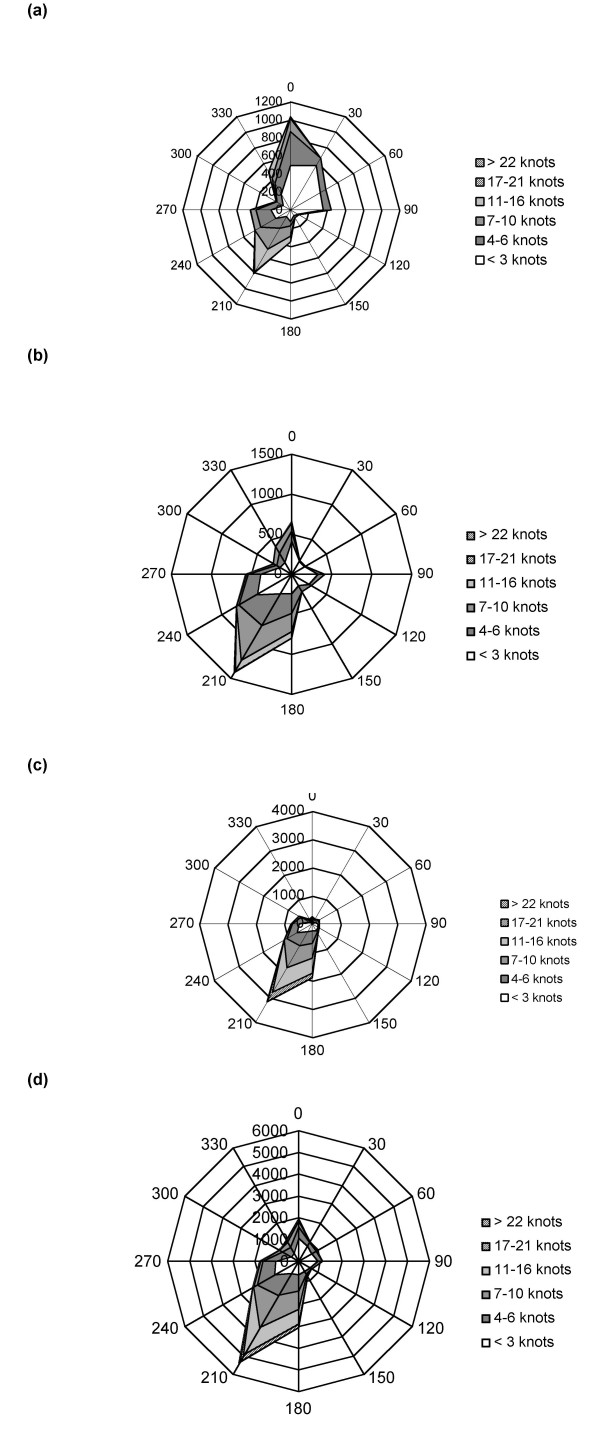
Wind rose illustrating speed (knots) and direction of the wind every 15 minutes y-axis represents the number of 15 minute occurrences with the x-axis's representing direction in degrees. (a) Sampling before the closure of the blast furnace (1/6/00 – 25/6/00). (b) During the closure of the blast furnace (26/7/00 – 28/9/00). (c) Sampling after the blast furnace reopened (29/9/00–31/12/00) and (d) the total sampling period (1/6/00–31/12/00).

Wind roses provided a visual aid when considering the effects of direction and speed. Although they can be constructed to display any period of time, the wind roses (Figure 3) prepared for the Redcar area refer to before (1/6/00 – 25/7/00), during (26/7/00–28/9/00) and after (29/9/00–31/12/00) the closure of the Corus blast furnace. A wind rose representing the whole period (June – December 2000) was also constructed.

The wind rose constructed for the three weeks between 1/6/00 and 25/7/00 covering the pre-closure sampling period (Figure 3a) showed that during this period the majority of wind came from a north-to-north easterly direction (approx. 0–30°), however a smaller proportion was also directed from the south west (approx. 210°). The wind speed coming from the north was generally below 6 knots and predominantly less than 3 knots, with some faster episodes of 7–10 knots and 11–16 knots. South-westerly winds did reach speeds of 11–16 knots, but most ranged from between 7–10 knots with some as low as 4–6 knots.

The wind rose constructed for the closure period (Figure 3b) indicates the majority of the wind came from the SW (approx. 210°). Wind does however come from the N to NW direction, although this is minimal when compared with the volume coming from the SW. The speed of SW winds ranged from less than 3 knots to 16 knots, but the wind speeds generally occurred between 4–10 knots, although slower speeds did take place more westerly (<3 knots).

For the sample period after the blast furnace reopened the wind rose (Figure 3c) indicates that the wind came solely from the SW and that the range of speeds recorded was from less than 3 knots to 21 knots, all of equal prominence.

The wind rose of Figure 3d covers all three time points discussed previously summarising wind speed and direction for the whole sampling period. The chart indicates that the majority of the wind came from the SW with speeds ranging from less than 3 knots to 21 knots; the most commonly recorded wind speeds fell within 4 – 16 knots. A relatively small fraction came from the N to NE direction at a wind speed predominantly less than 3 knots.

### PM_10 _atmospheric concentrations from sampling periods in years prior to, during and after the closure

Table 3 lists the maximum and the minimum 24 hour PM_10 _concentrations observed throughout the sampling period for 2000 and for the same period during 1999 and 2001. The lowest maximum and minimum mean daily PM_10 _concentrations occurred during the year the steel plant closed (46 μgm^-3 ^and 4 μgm^-3 ^respectively). The year before and after the closure of the plant saw maximum mean daily PM_10 _concentrations, exceeding the EU and UK 24 hour ambient concentration limit values of 50 μg/m^3^, that should not be exceeded more than 3 times in one year (Table 3).

**Table 3:** The daily mean PM_10 _concentrations (μgm^-3^) during the sampling period in 2000 the same periods in 1999 and 2001 for the Redcar and Cleveland area. (Data obtained from NETCEN).

	**Maximum PM_10 _Concentration μgm^-3^**		**Minimum PM_10 _Concentration μgm^-3^**	
Year	**Value**	**Date**	**Value**	**Date**

1999	50	06/09/99	6	27/09/99
2000	46	11/09/00	4	18/09/00
2001	52	11/12/01	5	12/08/01

### Redcar PM_10 _metals analysis

**Figure 4:**
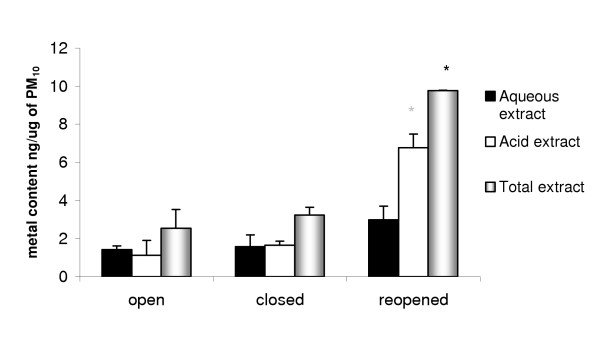
The measured metal content of 7 day PM_10 _samples collected before, during and after closure (* p < 0.05 compared to closure period). Extracts were made into ultra pure water (aqueous extract) followed by digestion of the remaining filter in HCl:HNO_3_(acid extract). Measurements were conducted by ICP-MS and values are the mean of 2 samples ± SEM.

The metal content of 7-day PM_10 _samples collected before, during and after the short-term closure of the Corus steel plant in Redcar was determined by ICP-MS. The PM_10 _samples were subjected to both aqueous and acid extraction sequentially as described in the methods. The combined results for both the aqueous and acid extractions were summed to give the total metal content of the PM_10 _samples. There was a significant increase in the total and acid extractable metal content of the PM_10_samples collected after the plant reopened when compared to that collected during the closure period (Figure 4). The aqueous extractable metal content did not differ significantly between the open and closed periods, although changes in specific transition metals did occur as, described below.

**Figure 5:**
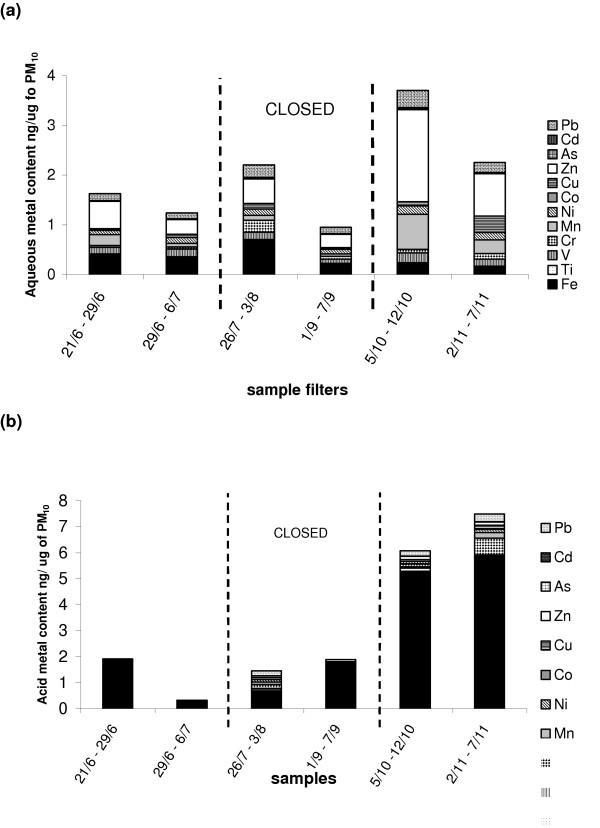
Metal content of PM_10 _collected before, during and after the closure of the Redcar Corus steel plant. (a) Aqueous extractable (b) acid extractable metal content of PM_10_. Measurements were conducted by ICP-MS and values are of individual filter samples

Figure 5a shows the aqueous extractable transition metal components of the same PM_10 _samples described above. The soluble iron content was considerably lower than the total iron content, indicating a substantial proportion of iron was insoluble. Furthermore the soluble iron content of PM_10 _did not significantly alter between the open and closed periods of collection. In contrast, soluble zinc, which occurs at notable levels in all samples, increased dramatically on reopening of the plant (1.86 ng/μg PM_10 _compared with 0.26 ng/μg PM_10 _during closure). In addition, both copper and manganese increased significantly on reopening when compared to the closure period (0.33 ng/μg PM_10 _compared to 0.03 ng/μg PM_10 _and 0.7 ng/μg PM_10 _compared with 0.05 ng/μg PM_10 _respectively). Figure 5b shows data collected from the acid digest of the filter and PM_10 _not removed by the aqueous extraction and hence represents mainly the insoluble metal components of the PM_10. _Iron was the most abundant of all the acid extractable metals analysed and increased greatly on reopening of the steel plant (5.81 ng/μg PM_10 _compared with 0.69 ng/μg PM_10_). As observed in the aqueous extract both copper and manganese increased significantly in the acid extract on reopening when compared to the closure period (0.15 ng/μg PM_10 _compared to 0.01 ng/μg PM_10 _and 0.22 ng/μg PM_10 _compared to 0.02 ng/μg PM_10_).

### Toxicology of Redcar PM_10_

**Figure 6:**
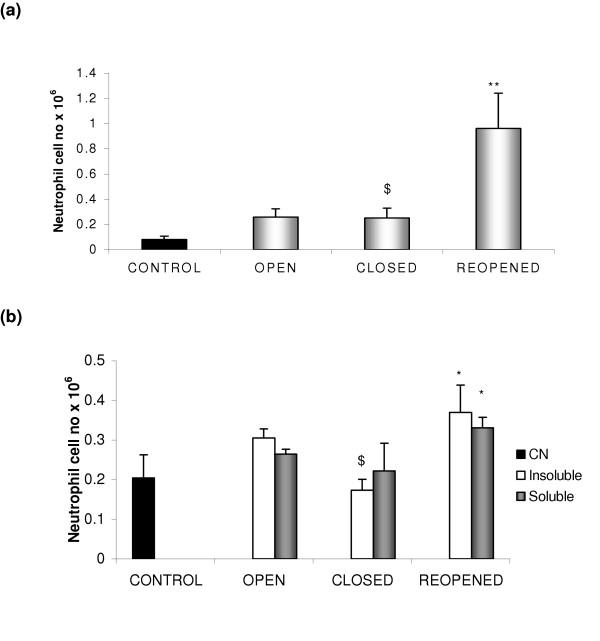
The mean neutrophil number in bronchoalveolar lavage from rat's 18 hr after exposure to either (a) total aqueous extracts or (b) the soluble or insoluble fractions of aqueous extracts of PM_10 _samples collected before, during and after the steel plant closure. Control animals were instilled with saline. Values represent the mean of (a) 6 and (b) 3 rats ± SEM (** p < 0.01 and * p < 0.05 to control, $ p < 0.05 closed to reopened

Samples of the same aqueous extracts of PM_10 _analysed by ICP-MS were subsequently instilled into male Wistar rats. The aqueous PM_10 _extracts taken before and during the closure did not alter significantly the total number of lavage cells recovered (table 4) nor did the aqueous extracts induce any significant increase in neutrophil content (neutrophil number or % neutrophils) of BAL when compared to the saline control (Figure 6a and table 4). However PM_10 _extracts from the reopened period induced a significant increase in neutrophil cell number and percentage neutrophils when compared to animals treated with the extracts of PM_10 _from the closed period or the control animals (Figure 6a and table 4).

**Table 4:** The mean bronchoalveolar lavage total cell count and percentage neutrophil influx ± SEM from rat's 18 hr after exposure to either total aqueous extracts or the soluble or insoluble fractions of aqueous extracts of PM_10 _samples collected before, during and after the steel plant closure. Control animals were instilled with saline.

**Treatment**	**Total cell number × 10^6 ^± SEM**			**% Neutrophils ± SEM**		
	*Aqueous*	*Soluble*	*Insoluble*	*Aqueous*	*Soluble*	*Insoluble*

**Control**	2.83 ± 0.34	3.85 ± 0.59	3.85 ± 0.59	3 ± 0.9	4 ± 0.9	4 ± 1.0
**Open**	3.40 ± 0.27	3.02 ± 0.16	4.16 ± 0.13	8 ± 1.8	9 ± 0.9	10 ± 0.9
**Closed**	3.06 ± 0.29	3.16 ± 0.18	2.63 ± 0.46	8 ± 1.0	10 ± 0.9	7 ± 1.0
**Reopened**	3.71 ± 0.38	2.85 ± 0.74	3.12 ± 0.17	25 ± 4	13 ± 1.5	10 ± 1.5

The soluble and insoluble extractable PM_10 _components that were washed from filters in the aqueous extract were separated by centrifugation and subsequently instilled into male Wistar rats. The soluble PM_10 _fraction of the extracts taken before and during the closure did not induce any significant changes in the number or percentage of neutrophils in BAL when compared to the saline control (figure 6b and table 4). However the water soluble fraction of aqueous PM_10 _extracts from the reopened period induced a significant increase in neutrophil cell number (p < 0.05) when compared to the control (Figure 6b and table 4). The insoluble fraction of PM_10 _washed from the filter taken before and during the closure did not induce any significant inflammogenic effect when compared to the saline control (Figure 6b and table 4). However the insoluble components of PM_10_extracts from the reopened period induced a significant increase in neutrophil cell number (p < 0.05) when compared to both the control and (p < 0.05) closed period samples (Figure 6b). The neutrophil cell numbers counted in BAL after treatment with the soluble and insoluble extracts from the reopened periods were each approximately half those obtained on treatment with the whole sample from the reopened period. In fact, these values when added together equalled the neutrophil influx measured for the total aqueous extract. However, the neutrophil values obtained for the insoluble and soluble exracts did not add up to equal the neutrophil response observed for the total aqueous extract as the increase in neutrophil influx was not significant for these periods.

Treatment of the rats with whole aqueous extracts of PM_10 _from any collection period did not significantly increase BAL content of MIP2 or TNFα when compared with the saline control (table 5). However, the overall trend of results are similar to those observed for the neutrophil cell count and the PM_10 _metals content, that is an increase in neutrophil and metal levels were observed when the plant was reopened compared with the closure period.

**Table 5:** Biochemical analysis of primary bronchoalveolar lavage fluid. Markers of inflammation measured included the chemokine MIP-2 and the cytokine TNFα. Total protein content, albumin and LDH were all measured Values are the means of 3 observations ± SEM.

	**Protein ug/ml**		**LDH U/ml**		**MIP2 pg/ml**		**TNFα pg/ml**	
**Control**	121.67	±28.67	236.75	± 55.42	22.52	± 22.52	2.20	± 2.20
**Open**	97.33	± 13.03	229.63	± 33.57	33.14	± 12.96	0.00	± 0.00
**Closed**	103.50	± 13.41	233.17	± 34.58	51.74	± 16.17	2.07	± 2.07
**Reopened**	114.83	± 13.20	263.66	± 34.33	80.61	± 29.99	6.84	± 3.17

**Figure 7:**
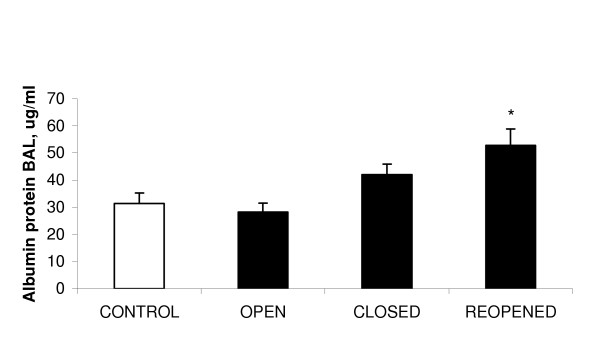
The mean concentration of albumin protein in BAL fluid from lungs exposed to aqueous extracts of PM_10 _and saline control (* p < 0.05 to control). Values represent the mean of 3 experiments ± SEM.

Markers of lung damage including total protein and LDH did not increase in the BAL fluid of rats exposed to the whole aqueous extract of PM_10 _for 18 hrs when compared to the saline instilled rats. In contrast, the albumin content of BAL fluid increased significantly in rats instilled with PM_10_collected when the steel plant reopened compared to the control animals (Figure 7).

**Figure 8:**
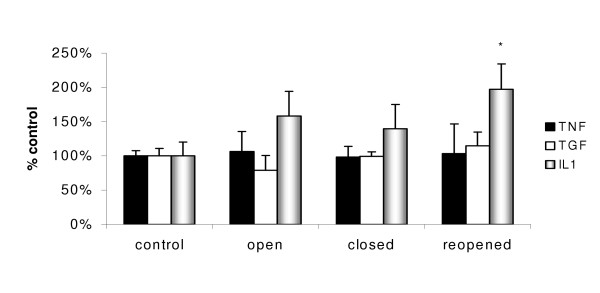
The mRNA expression by rat bronchoalveolar lavage cells 18 hr following instillation of aqueous extracts of PM_10 _collected before, during and after the steel plant closure (* p < 0.05 to control). Values represent the mean of 6 rats ± SEM.

The mRNA expression of a range of pro-inflammatory cytokines (IL1β, IL6, MIP2, TNFα, TGFβ and GM-CSF) by BAL cells was analysed in response to exposure of rats to either saline (control) or aqueous extracts of PM_10 _by RT-PCR. The PM_10 _collected during any period of steel plant operation did not alter the mRNA expression levels of the cytokine TNFα, and the pro-fibrotic and inflammatory cytokine TGFβ when compared with the control (Figure 8). In contrast, mRNA expression of the cytokine IL1β by BAL cells did increase significantly in rats instilled with extracts of PM_10 _obtained on reopening when compared with the control. The mRNA expression of IL1β exhibits a similar trend to that observed for the metals analysis (Figure 4) and neutrophil influx (Figure 6). The mRNA for IL6, MIP2 and GM-CSF were not detectable in the BAL cell extracts from either control or treated animals.

### Chelation of Redcar PM_10 _metals

J774.A1 cells were treated for 4 hrs with Redcar PM_10 _samples taken from during the closure and on reopening of the plant. Cells were also treated with identical PM_10 _samples that previously underwent treatment with Chelex beads for 4 hrs to remove metals from samples.

**Figure 9:**
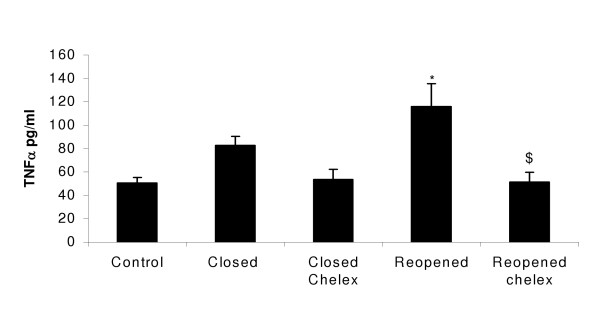
TNFα protein production in J774.A1 cells treated for 4 hrs with Redcar PM_10_sample during and after the closure of the Corus plant and identical samples which underwent 4 hrs of chelating treatment. *p < 0.05 when compared to control, $p < 0.05 when compared to untreated reopened PM_10 _samples. Values represent the mean of 3 experiments ± SEM.

Both the closed and reopened sample treatments stimulated the macrophage cells to increase TNFα protein release compared to control cells. Chelated closure PM_10 _sample treatments showed lower TNFα levels when compared with non-chelated treatments, almost returning to control levels (figure 9). Reopened chelated PM_10 _samples indicate a significantly lower TNFα production than non-chelated treatments, a significant decrease (p < 0.05) again almost returning to control levels (figure 9).

## Discussion

The PM_10 _sampling convention is mass-based and does not take account of composition. In this study the daily airborne mass of PM_10 _monitored by the AUN site before, during and after the steel plant closure did not alter significantly [13]. The samples studied had the greatest mass for the closure period. The ability of PM_10 _to induce inflammation in the rat lung when instilled varied greatly between the collection periods, with PM_10 _from the reopened period being most potent in causing inflammation despite the probability that this sample contained the lowest PM_10 _mass. The ability to cause pulmonary inflammation is generally considered to play an important role in the pulmonary and cardiovascular effects associated with increased PM exposure [14-16]. This study confirms the importance of composition in driving the pro-inflammatory effects of PM in this animal model, and by implication the adverse effects in exposed human populations. However, in this study it was not possible to determine the exact mass dose administered to each animal due to the low concentration of the samples employed. What is provided is an estimate of the maximum dose instilled per animal based upon the flow rate and the ambient PM_10_concentration. An identical extraction procedure was used for the PM_10 _samples collected from each sampling period, and this study assumes that the extraction efficiency is identical across all periods. Significant changes in particle composition however, could impact upon this process. However, an exact analysis of the metal content of each instilled sample was obtained, and as discussed below the metal content of the PM_10 _did impact directly on the potency of the sample instilled into each animal.

Data on the metal content of the PM_10 _showed that it was greatest in the 'reopened' sample, supporting the contention that this was indeed the factor that was responsible for the differences in ability to cause inflammation between the different PM samples. Study of the PM collected around a steel mill closure in Utah Valley, revealed a similar impact on composition. A greater inflammogenicity in rats exposed to the PM collected when the Utah plant was open was replicated in human lungs exposed by instillation [4,5].

The metal composition of PM_10 _samples varied, between the Redcar plant being operational and closed, in terms of both total metal content and concentrations of individual metals. The total metal content of PM_10_collected before the closure and during the closure was similar, but on reopening of the steel plant there was a 3-fold increase in total metal content of PM_10 _compared with the closure and pre-closure samples. This generally supports the hypothesis that, when the steel plant was closed, metal emissions were lower than during the operational period. The metal content of PM_10 _was relatively low before the closure of the plant but this may be a result of wind direction during the pre-closure sampling period, as the wind predominantly originated from the North Sea and hence not the Corus operations, in contrast to the closure and re-opened sampling periods. Although the change in metal content of the PM in relation to closure of the Corus plant may have occurred by chance and was a result of changes in emissions from other local sources, the emission inventory for this area shows such a remarkably predominant contribution from the Redcar steelworks that this seems unlikely [8].

The large increase in total metal on reopening of the steel plant was reflected in an increase in the acid extractable metals but not in the aqueous extractable metals, suggesting that the emissions from the steel plant contain metals which are predominantly water insoluble. However, within the total, acid and aqueous extracts of PM_10_, the individual metal contents varied significantly between the closed and reopened period. Of the twelve metals measured by ICP-MS, iron was the most abundant metal in both the total and acid-extractable fractions, in keeping with the nature of work being carried out at the plant. However in the aqueous extracts iron levels were small in comparison with other metals such as zinc and manganese, which again suggests that the iron particulate produced by the steel plant is predominantly insoluble. The metals which increased in the aqueous extract of PM_10 _on reopening of the steel plant included zinc, manganese and copper.

The same aqueous extracts analysed for metal content were also instilled into rats to determine the inflammogenic potency. The whole aqueous extractable Redcar PM_10 _samples collected prior to or during the steel plant closure, did not induce a significant increase in inflammation in the rat lung compared to the control. However whole PM_10 _collected on reopening of the plant, induced a significant increase in neutrophil influx into the lung. As discussed previously, the metals in PM_10 _that increased the most in the reopened period compared to the closed period were zinc, manganese and copper. Hence there was a clear relationship between metal content and inflammogenic potency of the PM_10 _samples. Metal chelation of the Redcar samples *in vitro *showed a significant decrease in pro-inflammatory protein production when compared to non-chelated treatments, re-affirming the suggested link between metal content and levels of inflammation.

When the soluble and insoluble sub-fractions of the PM_10 _collected during the reopened period were instilled, both samples induced neutrophil recruitment that was almost half that observed compared to the whole PM_10 _fraction from the same period. This would suggest that there are components in both the soluble and insoluble fraction that drive inflammation, and that they induced an additive effect in the rat lung. However, such an observation was not obvious for the pre-closure and closure period samples, possibly because none of these samples induced any significant increase in neutrophil influx compared to the control.

In addition to inducing inflammation, as indicated by cellular changes with in the lung, instillation with the whole aqueous extract of PM_10 _collected during the reopened period also induced a significant increase in mRNA expression of the pro-inflammatory cytokine IL1β in the BAL cells. In the same samples, the expression of TNFα and TGFβ mRNA expression did not change at the 18 hr time point investigated. Similarly, the chemokine MIP2 and pro-inflammatory cytokine TNFα protein content of BAL showed no statistical changes when compared with controls, but did exhibit similar trends to total metal content and neutrophil influx. The lack of significance was probably due to the dilution of BAL fluid during collection, as well as the use of a time point suitable for neutrophil influx but not TNFα measurement.

In agreement with previous work carried out in our laboratory, low doses of PM_10 _instilled into rat lungs did not induce indicators of gross cellular damage to lung cells (Lightbody *et al*., manuscript in preparation). However damage to the endothelium/epithelial barrier and hence an increase in the permeability of the vasculature was observed as indicated by an increase in the albumin content of the BAL fluid in rats exposed to PM_10 _extracts obtained on reopening of the steel plant. Hence, the PM_10 _samples that induced the greatest inflammation also induced the greatest lung damage and contained the highest metal content (especially for zinc, copper and manganese).

A number of recent studies have reported toxicity of metals and implicated them in the biological potency of ambient particulate matter [17-19]. Transition metal content of PM_10 _has been shown by Jimenez *et al*., [20] to activate transcription factors such as nuclear factor kappa B in epithelial cells, so linking transition metal content to the induction of inflammation. Wilson *et al*., [21] also showed that ultrafine carbon particles induced an inflammation in the rat lung that was potentiated by the addition of iron chloride, establishing an interaction between metals and particles in enhancing potency.

In conclusion, the PM_10_-induced inflammation was related to the concentration of metals in the PM_10 _samples tested and the reopening of the steel plant was associated with an increase in the metal content of the PM sufficient to change an average 180 μg/ml instilled dose from non-inflammogenic to inflammogenic. It is unlikely that all of the measured metals were biologically active in the Redcar PM_10 _samples, with some associations being correlative rather than causative. The potency of the biologically active metals is likely to vary, for example, Riley *et al*.[22] was able to rank metal toxicity to rat lung epithelial cells (V>Zn>Cu>Ni>Fe) by measuring TC50. Rice *et al*., [18] also showed that instillation of soluble metals into the rat lung induced inflammation. Copper was the most pro-inflammatory metal in their study followed by manganese and nickel, while vanadium, iron and zinc induced similar levels of inflammation. This study analysed the metal content of Redcar PM_10 _and related composition to toxicological effects observed. The effects of elemental carbon, organic compounds and biological components cannot be ignored, however no data on whether organic components of PM actually changed during the course of this study are available.

In conclusion, this study indicates that the operations of a local UK Steel plant impacts significantly on the metal content of PM_10_. Furthermore, this study confirms previous observations that the metal composition of PM_10 _is related to its ability to drive inflammation in the rat lung. This was confirmed by the ability of metal chelation to block the *in vitro *effects of the metal rich PM_10 _samples. Such observations are important when considering the potential impact on the health of susceptible people living within the vicinity of such industrial operations.
